# Capillary Bronchitis, with Brain Symptoms, Cured with Stramonium

**Published:** 1895-10

**Authors:** Samuel A. Kimball

**Affiliations:** Boston, Mass.


### CAPILLARY BRONCHITIS, WITH BRAIN SYMPTOMS, CURED WITH STRAMONIUM.

SAMUEL A. KIMBALL, M. D., BOSTON, MASS.

F. B., a little girl three and one-half years old, had been ailing for a few days with a cough and some disturbance of the digestive tract, with pain in the abdomen at night after eating, much loud belching and offensive flatus. She seemed better, and was allowed to go out, but her cough became worse, and I was telephoned late one night that she was in great pain, evidently in the abdomen, sharp pains coming and going suddenly, and causing her to cry out, much rumbling of wind, hot skin, restlessness, with twitching of muscles and of limbs in sleep.

I advised them to give Bell.$^{1m}$ in water for three doses unless relieved.

In the morning I found her no better, and there was a much more serious condition present than the parents or I had been aware of.

She was delirious, moaning, crying out, and sometimes singing; there were twitching of the muscles of the face, twitching of the lips, making faces, distortion of the face, grinding of teeth, twitching of fingers, dilated pupils; a hard, racking cough, with involuntary urine at times, rattling respiration and fine râles all over the chest, front, and back. There was a desire for light, but not marked. The skin was very hot. Pulse, 160; respiration, 84.

The important symptoms seemed to be the moaning, singing, crying out, grinding of the teeth, and distortion of face.

Singing and moaning give: Apis, Bell., Cic., Cocc., Cupr., Hyosc., Lach., Mag-c., Nat-c., Op., Phos., Sep., Stram., Tabac., Tarent., Sul-ac.

Grinding of teeth gives with these: Apis, Bell., Cic., Hyosc., Phos., Sep., Stram.

Distortion of face and crying out: Apis, Bell., Cic., Hyosc., Stram.

The desire for light would narrow it down to Bell. and Stram.; but carefully comparing the five preceding remedies, Apis, Bell., Cic., Hyos., and Stram., Stramonium seemed best suited to the case, covering the twitchings, the dilated pupils, rattling respiration, and râles in the chest, and while it does not have involuntary urine with cough, it has it with other conditions.

One dose of Stramonium$^{cm}$ (F.) was given dry at 10.30 A. M.

At 3 P. M. they reported at my office that the conditions were the same, no better; if anything, a little worse.

In a case with so sudden an onset of serious symptoms it seemed as if the remedy should have accomplished something in that length of time, and Stramonium seemed still the best indicated remedy. The child, however, did not usually respond well to remedies, and I sent one dose of Sulphur$^{200}$ to be given dry.

At 6 P. M. I found her better. There had been intervals of quiet, less crying out, less moaning, and she had slept quietly for half an hour; but there was much grinding of the teeth, and she had mewed like a cat several times.

There was one peculiarity about her condition. She would repeat for a long time any outside noise that she heard, such as the sound of a drum, mewing of a cat, etc. This she would do over and over again. No medicine was given.

At 9 P. M. she was worse. All the original symptoms had returned with increased violence; there had been but little sleep, and twice she had arched her body, resting on her head and heels, almost a condition of opisthotonos.

There were the same grinding of the teeth, distortions of the face, crying out, singing and moaning.

It seemed still a Stramonium condition, and Stramonium$^{200}$ was dissolved in water and a dose given at 9.30 P. M., with instructions to repeat every hour for three doses if necessary, but to stop if there was any improvement.

The next morning I found her much better. After the first dose at 9.30 the night before there was an improvement. She slept some and was more quiet until midnight, when there was a repetition of the moaning, crying out, and twitching of the face, but after another dose she was quiet the remainder of the night and slept for some time. She had taken milk and was rational in the morning.

There was still rattling respiration, and cough. Pulse, 144; respiration, 72. She preferred to lie upon the right side. Her father, an excellent observer, told me he frequently counted her respirations during the night as 120 per minute. No medicine.

In the evening she was still improving, rational, no more twitchings or crying out, and the respirations had fallen to between 48 and 60.

The next morning I found she had passed a good night, the cough was better, and the respirations were from 40 to 45. She rapidly progressed to a complete recovery, and, as is often the case, was much better than for a long time.

In looking over the case, would it have been better to have given the Stramonium$^{200}$ in water at first, or to have repeated it in water after the failure of the CM dry without giving the Sulphur?

Such speculations are always interesting, but a successful result is apt to bias our opinion in favor of what was done. Possibly, repeated doses might have been successful at first, but in such severe cases of nervous excitability the single dose, high, is usually to be preferred, especially in children, who may be extremely susceptible to repetitions, and a severe aggravation result.

When this single dose failed, would it have been better to repeat the same, or another potency, in water?

If the Stramonium$^{cm}$ was the remedy and potency, it seems as if some amelioration ought to have shown itself, if only for a short time; but there seemed to be no response, and on account of her former lack of reaction to remedies the Sulphur was given. When the Stramonium was given again there was no doubt about the remedy; the only question was what potency to give and how to give it, and the 200th was selected and repeated in water.

Adjourned to 2 P. M.

---

SECOND DAY. AFTERNOON SESSION.

Thursday, June 27th, 2 P. M.

The following telegram was received from Dr. Adams, of Toronto, who was prevented by illness from attending the meeting:

"Though absent, yet present. May you have a medical Pentecost. Greeting to all.

"ADAMS."

Dr. J. W. Thomson presented the following amendment in writing:

"*Resolved*, That the words 'spirit-like life-force' be stricken from sections I and III of the Declaration of Principles, and that the words 'vital force' be used in their stead."

